# Approaching the standard quantum limit of mechanical torque sensing

**DOI:** 10.1038/ncomms13165

**Published:** 2016-10-20

**Authors:** P. H. Kim, B. D. Hauer, C. Doolin, F. Souris, J. P. Davis

**Affiliations:** 1 Department of Physics, University of Alberta, CCIS 3-199, Edmonton, Alberta, Canada T6G 2E9

## Abstract

Reducing the moment of inertia improves the sensitivity of a mechanically based torque sensor, the parallel of reducing the mass of a force sensor, yet the correspondingly small displacements can be difficult to measure. To resolve this, we incorporate cavity optomechanics, which involves co-localizing an optical and mechanical resonance. With the resulting enhanced readout, cavity-optomechanical torque sensors are now limited only by thermal noise. Further progress requires thermalizing such sensors to low temperatures, where sensitivity limitations are instead imposed by quantum noise. Here, by cooling a cavity-optomechanical torque sensor to 25 mK, we demonstrate a torque sensitivity of 2.9 yNm/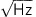. At just over a factor of ten above its quantum-limited sensitivity, such cryogenic optomechanical torque sensors will enable both static and dynamic measurements of integrated samples at the level of a few hundred spins.

## Abstract (web summary)

Cavity optomechanics enables measurement of torque at levels unattainable by previous techniques, but the main obstacle to improved sensitivity is thermal noise. Here the authors present cryogenic measurement of a cavity-optomechanical torsional resonator with unprecedented torque sensitivity of 2.9 yNm/√Hz.

Mechanical transduction of torque has been key to probing a number of physical phenomena, such as gravity^1^, the angular momentum of light^2^, the Casimir effect^3^, magnetism^4^,^5^ and quantum oscillations^6^. Following similar trends as mass^7^ and force^8^ sensing, mechanical torque sensitivity can be dramatically improved by scaling down the physical dimensions, and therefore moment of inertia, of a torsional spring^4^. To measure the tiny mechanical displacements associated with nanoscale torsional resonators, it is now possible to turn to the new field of cavity optomechanics^9^. Cavity optomechanics allows measurement of extremely small mechanical vibrations via effective path length changes of an optical resonator, as epitomized by the extraordinary detection of the strain resulting from transient gravitational waves at LIGO^10^. Harnessing cavity optomechanics has enabled measurements of displacement (the basis for force and torque sensors) of on-chip mechanical devices^11^,^12^ at levels unattainable by previous techniques. Applied to torsional measurements, cavity optomechanics has enabled us to reach the point^13^,^14^ where geometric optimization of the moment of inertia can only provide marginal improvements to torque sensitivity. Instead, nanoscale optomechanical measurements of torque are overwhelmingly hindered by thermal noise.

Fortunately, cavity optomechanics has been successfully integrated into cryogenic environments. Multiple architectures are now capable of cooling near the quantum ground state either directly through passive cooling^15^,^16^,^17^, or in combination with optomechanical back-action cooling^18^,^19^,^20^. Yet, only passive cooling is compatible with reducing the thermal noise of an optomechanical torque sensor. In addition, although these architectures are well suited to tests of quantum mechanics and applications of quantum information processing, they are not well suited to integration with external systems one may wish to test.

Here we present measurements of a cavity-optomechanical torsional resonator with a room temperature torque sensitivity of 0.4 zNm/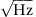, improved over previous generations of devices^13^,^14^. When cooled in a dilution refrigerator to a temperature of 25 mK, corresponding to an average phonon occupation of 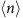
=35, this device demonstrates a record-breaking torque sensitivity of 2.9 yNm/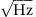. This represents a 270-fold improvement over previous optomechanical torque sensors^13^,^14^ and is just over an order of magnitude from its standard quantum limit (SQL). Furthermore, we demonstrate that mesoscopic test samples such as micrometre-scale superconducting disks^21^ can be integrated with our cryogenic optomechanical torque-sensing platform, in contrast to other cryogenic optomechanical devices^16^,^17^,^18^,^20^, opening the door for mechanical torque spectroscopy^22^ of intrinsically quantum systems. Hence, our cavity optomechanical torque-sensing platform is unique, in that it enables straightforward integration with test samples and operates in a dilution refrigerator with near quantum-limited torque sensitivity.

## Results

### Limits of torque sensitivity

For a thermally limited classical measurement, the minimum resolvable mechanical torque as inferred from the single-sided, angular displacement spectrum (see Supplementary Notes 1 and 2) is given by


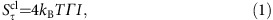


where *k*_B_ is the Boltzmann constant and *T* is the mechanical mode temperature. By taking the square root of equation (1), one obtains the device's torque sensitivity (in units of Nm/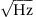). Minimization of the (effective) moment of inertia, *I*, (see Supplementary Note 3) and the mechanical damping rate, *Γ*, can therefore result in improved torque sensitivity at a given temperature. Reducing the mechanical damping is notoriously challenging and even with modern nanofabrication techniques—paired with cavity optomechanical detection of sub-optical wavelength structures—the moment of inertia can only be lowered so far. Therefore, further improvement demands lowering of the mechanical mode temperature.

To understand the limit of torque sensitivity in the quantum regime, one must consider the device's intrinsic angular displacement spectrum, 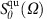, as well as the imprecision and back-action noise spectra associated with the measurement apparatus, 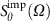 and 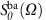, giving a total measured angular noise spectral density of


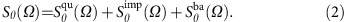


The intrinsic noise spectrum can be expressed as 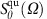
=|*χ*(*Ω*)|^2^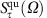, where *χ*(*Ω*) is the torsional susceptibility, and we introduce the quantum thermal torque spectrum, 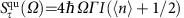 (see Supplementary Note 4). This spectrum is comprised of both the average phonon occupation of the mechanical mode, 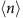, which for a resonator in equilibrium with an environmental bath is given by 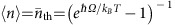, as well as a ground-state contribution, manifest as an addition of one-half to the resonator's phonon occupation.

Furthermore, the back-action and imprecision noise will result from a combination of both technical and fundamental noise associated with the measurement apparatus, whose product is bounded from below (for single-sided spectra^23^) by the Heisenberg uncertainty relation


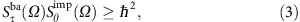


with equality corresponding to a measurement limited solely by quantum noise^24^. Using this relation, it is possible to determine the measurement strength at which the added fundamental back-action and imprecision noise will be minimized, corresponding to the SQL of continuous linear measurement. Considering a torque at the resonance frequency, *Ω*_m_, of the mechanical system—where the displacement signal is maximized—the minimum resolvable torque at the SQL is found to be


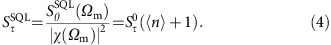


Here, 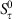
=4*ħΩ*_m_*ΓI* is the fundamental torque noise limit associated with a continuous linear measurement of a mechanical resonator in its ground state. Half of this fundamental torque noise arises from the zero-point motion of the resonator, the other half from the Heisenberg-limited measurement noise at the SQL. It is noteworthy that in the classical limit, 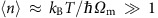, both of these effects can be neglected and the minimized quantum torque noise of equation (4) is equivalent to its classical counterpart given by equation (1).

### Passive versus active cooling

As illustrated by equation (4), the torque sensitivity is improved as the average phonon occupancy of the device is decreased. Therefore, one might naively think to use some form of active cold damping to reduce the phonon occupancy of the mechanical resonator and, consequently, its minimum resolvable torque. For example, one could employ optomechanical back-action cooling (OBC), which has been successful in reducing the phonon occupancy of nanoscale mechanical resonators near to their quantum ground state^18^,^19^,^20^. In OBC, a dynamical radiation pressure force imparted by photons confined to an optical cavity effectively damps the device's motion, increasing its intrinsic mechanical linewidth by an amount Γ_OM_ and reducing its average phonon occupancy according to


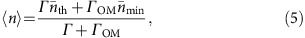


where 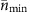 is the minimum obtainable average phonon number using this method^9^. Inputting this expression for 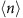 into equation (4), one can determine the minimum resolvable torque associated with OBC (see Supplementary Note 5) as


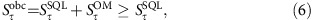


where 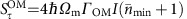 is the contribution to the minimum resolvable torque resulting from optomechanical back-action, physically manifest as fluctuations in the radiation pressure force of the cooling laser due to photon shot noise. Therefore, OBC has the counterintuitive effect of increasing the minimum resolvable torque spectrum, as any reduction in phonon occupancy of the resonator is negated by an accompanying increase in the mechanical damping rate. Thus, a torsional optomechanical resonator must be passively cooled towards ground-state occupation, to reach its SQL of torque sensitivity.

### Experimental system

**Figure 1:**
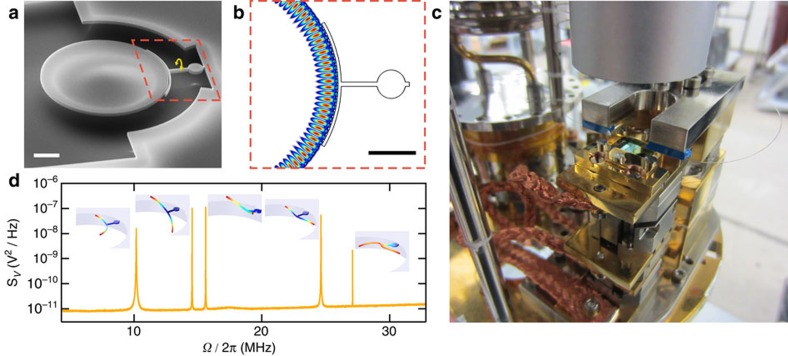
Low-temperature optomechanics. (**a**) Tilted-view scanning electron micrograph of the optomechanical torque sensor used here: a 10 μm diameter optical microdisk evanescently coupled to a torsional nanomechanical resonator by a vacuum gap of 60 nm. The torsional mode twists about the torsion axis, as indicated by the yellow arrow. Scale bar, 2 μm. A 1.1 μm diameter ‘landing pad' on the torsion rod allows for deposition of secondary test samples. (**b**) Finite element model of the microdisk optical resonance, which couples dispersively to the mechanical resonator. Scale bar, 2 μm and the red dashed box shows the same area as in **a**. (**c**) The sample chip is clamped into a gold-plated copper mount, on a stack of low-temperature-compatible nanopositioners, with thermal braids linking it to the base-plate of the dilution refrigerator^25^. A dimpled-tapered optical fibre is held on a positionable Invar fork above the sample stage and the chip-fibre system can be imaged by a low-temperature endoscope^25^. The dimpled-tapered fibre is used to excite the microdisk's optical mode and read out the optomechanical signal resulting from the Brownian motion of the mechanical resonances. (**d**) Optomechanically measured thermal noise voltage spectrum of the five lowest-order mechanical modes, at 4.2 K, with finite element simulations of the mode shapes colour coded by their total displacements. The second resonance, at 14.5 MHz, corresponds to the torsion mode with optimized signal to noise.

The optomechanically-detected torsional nanomechanical resonator operating inside a dilution refrigerator, at bath temperatures down to 17 mK, is shown in Fig. 1. Details of the cryogenic optomechanical system can be found elsewhere^25^. One merit of this system is that the use of a dimpled-tapered optical fibre^26^ results in a full system optical detection efficiency of *η*=32%, which aids in the low-power optical measurements described below.

The torsional mechanical mode (detailed in Supplementary Figs 1 and 2, as well as in Supplementary Table 1), at *Ω*_m_/2*π*=14.5 MHz, has low effective mass^23^, *m*=123 fg (geometric mass of 1.14 pg), low effective moment of inertia, *I*=774 fg·μm^2^, and low mechanical dissipation, *Γ*/2*π*=340 Hz. To sensitively measure the small angular motion of the device, we engineer a large angular (linear) dispersive optomechanical coupling, *G*_*θ*_=d*ω*_c_/d*θ* (*G*_*x*_=d*ω*_c_/d*x*—see Supplementary Note 6). The arms of the torsional resonator arc along the optical disk, over one-sixth of its perimeter, resulting in *G*_*θ*_=3.4 GHz mrad^−1^ (*G*_*x*_=1.4 GHz nm^−1^), while separating the mechanical element from the bulk of the optical field as compared with optomechanical crystals^11^,^16^,^17^. In addition, the optical resonance at *ω*_c_/2*π*=187 THz, the modeshape of which is shown in Fig. 1b, is over-coupled to the dimpled-tapered fibre, with external and internal loss rates *κ*_e_/2*π*=7.1 GHz and *κ*_i_/2*π*=2.6 GHz, further reducing optical absorption in the mechanical element. Owing to the small mass and low frequency of the torsional mode, its zero-point motion is relatively large with *θ*_zpf_=27 nrad (*x*_zpf_=69 fm), resulting in a single-phonon coupling rate of *g*_0_/2*π*=15 kHz and a single photon cooperativity of *C*_0_=3 × 10^−4^.

### Cryogenic measurements

**Figure 2:**
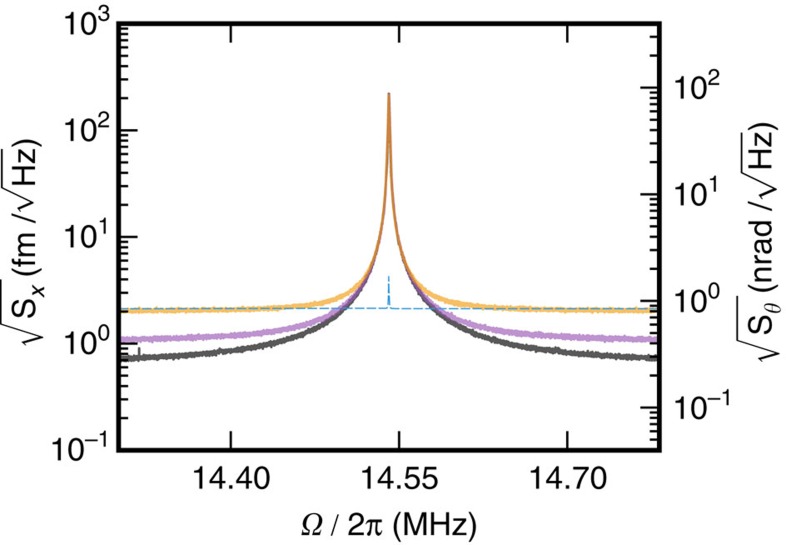
Power dependence at 4.2 K. Optomechanically measured thermal noise spectra, calibrated in terms of linear and angular displacement, of the torsional mode in the presence of helium exchange gas. As the optical power injected into the device is increased, the photon shot noise is reduced and the measurement imprecision drops below the noise floor of 0.84 nrad/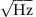 (2.1 fm/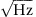) corresponding to the SQL. Optical power input to the device (average intracavity photon number) corresponds to: orange 33 μW (6.0 × 10^3^ photons), purple 86 μW (1.3 × 10^4^ photons) and grey 156 μW (2.7 × 10^4^ photons), resulting in angular (linear) displacement imprecision noise floors of 0.75 nrad/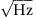 (1.9 fm/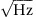), 0.39 nrad/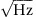 (0.97 fm/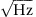) and 0.25 nrad/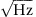 (0.62 fm/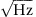). The zero-point noise spectrum at the SQL, calculated using measured device parameters, is shown as the blue dashed line.

**Figure 3:**
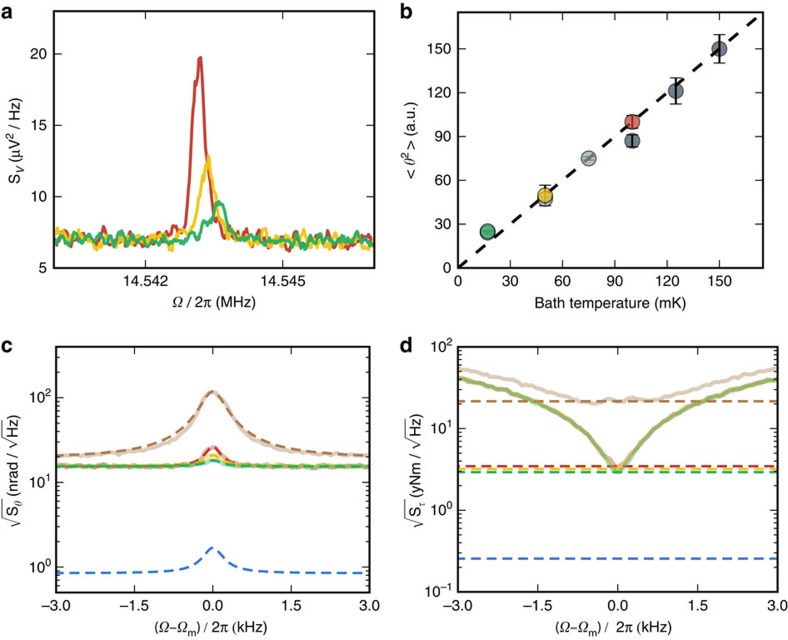
Data at mK temperatures. (**a**) Power spectral densities of the torsional resonance at 100 mK (red), 50 mK (yellow) and 17 mK (green) dilution refrigerator temperatures. To minimize optical heating, we use low input power at the device (1.26 μW—corresponding to ∼200 intracavity photons) and a low duty cycle measurement (see Supplementary Note 7 and Supplementary Fig. 3). (**b**) The integrated area under the power spectral density for three data runs, normalized to the highest temperature in each run. The integrated area under the power spectral density for three data runs, normalized to the highest temperature in each run. Error bars were calculated by varying the integration window by plus and minus 600s (corresponding to plus and minus 5% of the total integration time) and finding the resulting deviation in the integrated area. Slow system drift limits data acquisition to three temperatures per run. Slow system drift limits data acquisition to three temperatures per run. Coloured data points correspond to the measurements in **a**, whereas the light and dark grey points are additional runs. Linearity between the integrated areas and the bath temperature implies adequate mode thermalization down to 25 mK, corresponding to an average phonon occupancy of 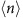
=35. The dashed line has slope unity, representing a well-thermalized mode. (**c**) Calibrated angular displacement spectral densities of the torsional mode at cryogenic temperatures (4.2 K brown, 100 mK red, 50 mK yellow and 25 mK green) with fits to equation (2) as dashed lines, along with the spectrum corresponding to zero-point fluctuations at the SQL calculated from device parameters in blue. The measurement imprecision is higher than at the SQL due to the low optical power used to limit heating. (**d**) Calibrated torque sensitivities corresponding to the measurements in **a**,**c**. Dashed lines are the resonant torque sensitivity, reaching 2.9 yNm/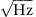 at *T*=25 mK: just over a factor of 10 above its quantum-limited value of 0.26 yNm/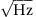.

First, we find that using helium exchange gas to thermalize optomechanical resonators to 4.2 K is effective, even at high optical input powers^27^,^28^, enabling measurement imprecision below the SQL as shown in Fig. 2. On the other hand, when the exchange gas is removed for operation at millikelvin temperatures, light injected into the optomechanical resonator causes heating of the mechanical element^16^,^17^,^28^. To combat this parasitic, optically-induced mechanical heating, we lower the duty cycle of the optomechanical measurement (see Supplementary Note 7). Using a voltage-controlled variable optical attenuator, we apply a 20 ms optical pulse (limited by the mechanical linewidth), while continuously acquiring AC time-domain data, which are subsequently Fourier transformed to obtain mechanical spectra. We then wait 120 s (corresponding to a duty cycle of 0.017%) for the mechanical mode to re-thermalize to the bath. To acquire sufficient signal-to-noise, this optical pulse sequence is repeated 100 times. The resulting averaged power spectral densities, as shown in Fig. 3a, are fit^23^ to extract the area under the mechanical resonance, 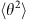, which is proportional to the device temperature. The mechanical mode temperature is confirmed by comparing the linearity of 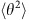 with respect to the mixing chamber temperature, as measured by a fast ruthenium oxide thermometer referenced to a ^60^Co primary thermometer (Fig. 3b). We find that the mechanical mode is well thermalized to the bath, except near the fridge base temperature of 17 mK at which point the mechanical mode temperature is limited to 25 mK, corresponding to an average phonon occupancy of 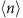
=35.

## Discussion

According to equation (4), 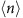
=35 corresponds to a measured torque sensitivity a factor of six above the fundamental quantum torque sensitivity limit, when operating at the SQL. Yet, thermomechanical calibration of the displacement using the mechanical mode temperature reveals that the low optical powers used to limit heating result in measurement imprecision above the SQL, as seen in Fig. 3c. Specifically, we find that we have 90 quanta of added noise—instead of the ideal single quantum—dominated by measurement imprecision, placing the measured torque sensitivity 11 times above its SQL of 0.26 yNm/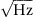. Nonetheless, calibration in terms of torque sensitivity, as seen in Fig. 3d, reveals that at 25 mK we reach 2.9 yNm/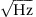, a 270-fold improvement over previous generations of optomechanical torque sensors^13^,^14^ and more than a 130-fold improvement from the room-temperature sensitivity of the same device.

**Figure 4:**
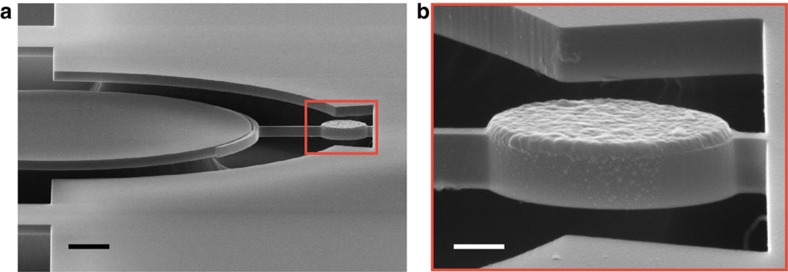
Torsional optomechanical resonator with integrated micrometre-scale aluminum disk. (**a**) Fabrication demonstration of the optomechanical torque-sensing platform via integration of a single mesoscopic aluminum disk (1.1 μm diameter, 45 nm thick). Scale bar, 1 μm. (**b**) Zoom-in around the aluminum disk with a scale bar of 250 nm. An optomechanical torque sensitivity of 2.9 yNm/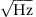 will not only enable measurements of single superconducting vortices^21^, but also for the first time the mechanically detected dynamics^22^ of single vortices. Room-temperature measurements show that the optical and mechanical modes are unaffected by the presence of the metallic test sample.

To give some idea of a what a torque sensitivity of 2.9 yNm/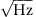 enables, imagine the experimental scenario described in refs 4, 22, where the torsional mode is driven on resonance by a torque generated from an AC magnetic field, **H**, orthogonal to both the magnetic moment of the test sample, **μ**, and the torsion axis, leading to **τ**=**μ** × **H**. A reasonable drive field of 1 kA m^−1^ would imply the ability to resolve ∼230 single electron spins within a 1 Hz measurement bandwidth. Excitingly, this sensitivity unleashes the advanced toolbox of mechanical torque spectroscopy^4^,^22^,^29^ on mesoscopic superconductors, previously limited to static measurements of bulk magnetization via ballistic Hall bars with ∼10^3^ electron spin sensitivity^21^. As a first step in this direction, we show in Fig. 4a prototype optomechanical torsional resonator with a single micrometre-scale aluminum disk integrated onto its landing pad, fabricated via secondary post-release e-beam lithography^30^. Room-temperature optomechanical measurements of devices with integrated aluminum disks in this geometry reveal that neither the optics or mechanics are degraded by the presence of the metal. Future integration of bias and drive fields into our cryogenic system will enable measurements of the dynamical modes^22^ of single superconducting vortices^21^, and even the paramagnetic resonance of electrons trapped within the silicon device itself.

## Methods

### Device fabrication

The device was fabricated from silicon-on-insulator (silicon thickness of 250 nm on a 3 μm buried oxide) using ZEP-520a e-beam resist on a RAITH150-TWO 30 kV system, followed by an SF_6_ reactive-ion etch to transfer the pattern to the silicon-on-insulator. The device was released from the oxide by 26 min of buffered-oxide-etch wet-etch followed by a dilute HF dip for 1 min and subsequent critical-point drying.

The prototype shown in Fig. 4 was fabricated to test the optical and mechanical effects of an Al film on the landing pad. Here the same nanofabrication procedure is followed, including the buffered-oxide-etch wet-etch, yet instead of drying the chip it was transferred to an acetone bath. The procedure for post-alignment then follows ref. 30: polymethylmethacrylate (PMMA) A8 resist was added to the acetone droplet on the chip to replace the acetone with resist. After spinning the resist, write-field alignments were done using pre-patterned alignment marks. High-purity aluminum (99.9999%) was deposited using an e-gun evaporation system at 2 × 10^−7^ Torr. After depositing 45 nm of Al, lift-off was performed by soaking in an NMP (*N*-methyl-2-pyrrolidone) solvent for 1 h at 80 °C. Afterwards, the chip was transferred to isopropyl alcohol (IPA) and then critical-point dried.

### Optomechanical measurements

Optomechanical measurements in the dilution refrigerator are performed as in refs 25, 28, yet using a dimpled tapered fibre^26^ with a ∼50 μm diameter dimple, and the pulse scheme described in Supplementary Note 7.

### Data availability

All relevant data are available from the authors upon request.

## Additional information

**How to cite this article:** Kim, P. H. *et al*. Approaching the standard quantum limit of mechanical torque sensing. *Nat. Commun.* **7,** 13165 doi: 10.1038/ncomms13165 (2016).

## Supplementary Material

Supplementary Information Supplementary Figures 1-3, Supplementary Table 1, Supplementary Notes 1-7 and Supplementary References.
