# Supplementary material for: Approaching the standard quantum limit of mechanical torque sensing
Source: Nat Commun. 2016 Oct 20;7:13165. doi: 10.1038/ncomms13165 (PMC5080439; doi:10.1038/ncomms13165)
Supplement: Supplementary Information — Supplementary Figures 1-3, Supplementary Table 1, Supplementary Notes 1-7 and Supplementary References. [file ncomms13165-s1.pdf]

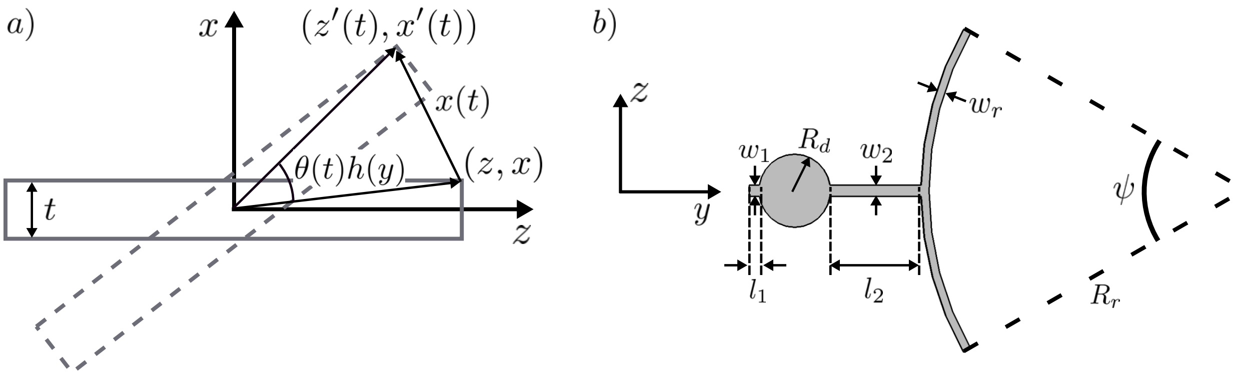

Supplementary Figure 1. **Out-of-plane torsional motion and resonator dimensions**. **a**, A schematic depicting the out-of-plane displacement of a simple torsional mode. **b**, A top-down view of the torsional resonator geometry used in this work with the critical dimensions labeled. The measured numerical value for each dimension can be found in Supplementary Table 1.

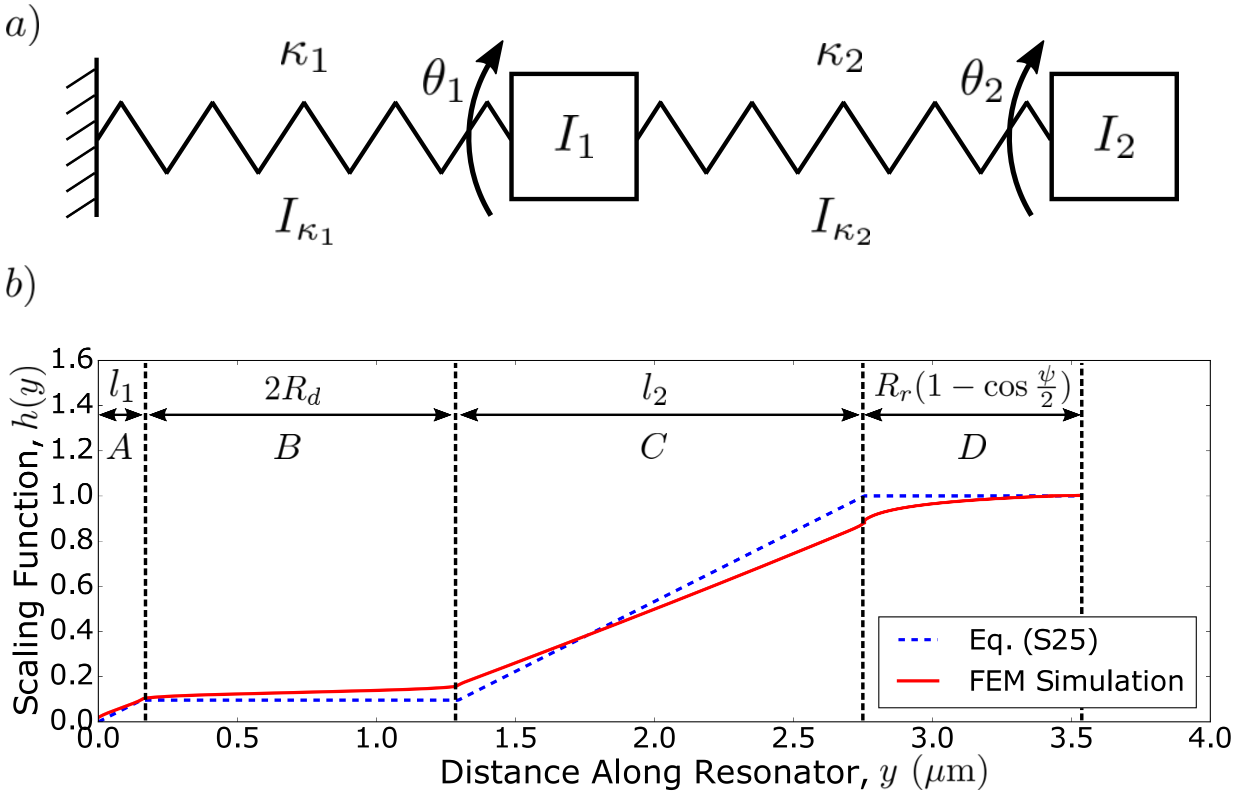

Supplementary Figure 2. **Coupled torsional model schematic and angular scaling function**. **a**, A torsional "mass-and-spring" diagram illustrating the simple coupled oscillator model used in Supplementary Note 4. **b**, Plot of the scaling function $h(y)$ using both the analytical model of equation (25) (blue - dashed) and FEM simulation (red - solid), with the following four regions of the resonator demarcated: *A* - first torsion rod, *B* - sample disk, *C* - second torsion rod and *D* - ring segment.

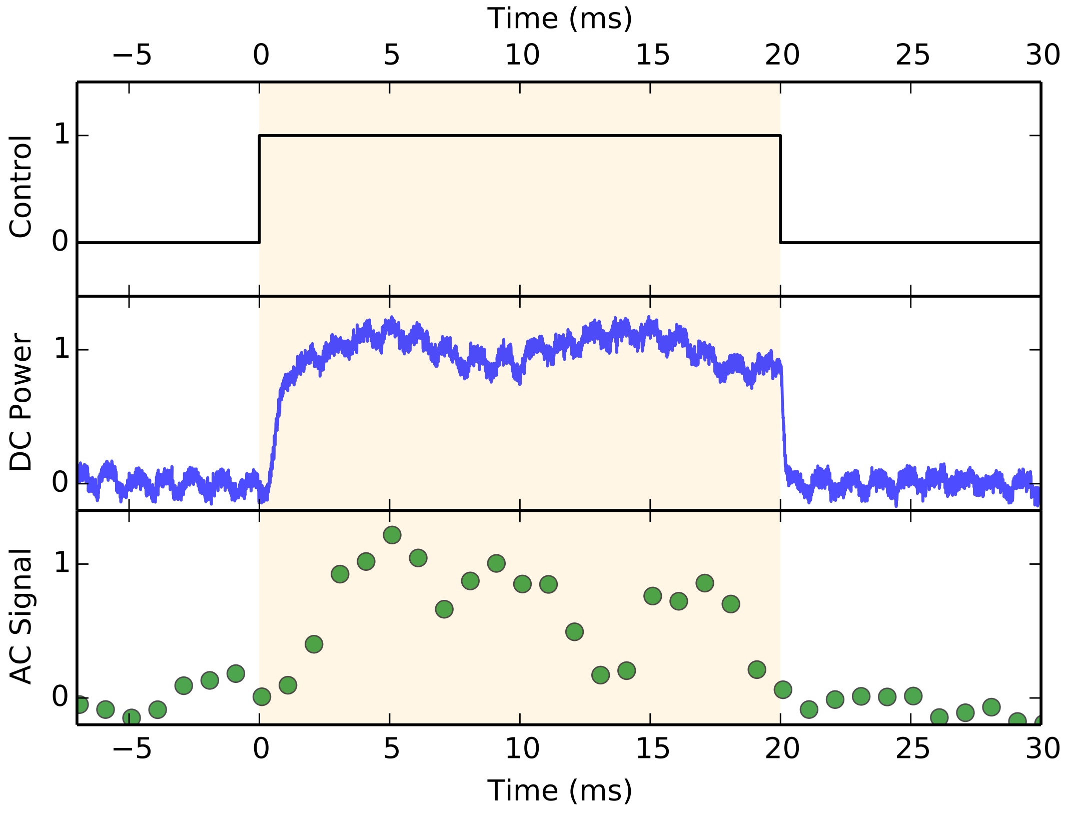

Supplementary Figure 3. **Low duty cycle measurements**. Light transmitted through the system is split into a DC component, which indicates the power in the optomechanical resonator, and an AC component. The AC signal is bandpassed over a 1.4 kHz bandwidth centered at the mechanical resonance frequency. The noise floor is estimated from 2 MHz of off-resonance signal and subtracted from the AC signal. Here we show this AC signal sliding-averaged across 4.2 ms for every 1 ms.

Measured Parameters	Material Parameters (Si)	Calculated Quantities
l_1 = 155 nm	$\rho = 2329$ kg·m^3	$\kappa_1 = 1.09 \times 10^{-10}$ N·m
w_1 = 175 nm	$E = 170$ GPa	$\kappa_2 = 1.16 \times 10^{-11}$ N·m
l_2 = 1.46 μm	$\nu = 0.28$	$I_{\kappa_1} = 1.23 \times 10^{-31}$ kg·m^2
w_2 = 175 nm		$I_{\kappa_2} = 1.15 \times 10^{-30}$ kg·m^2
w_r = 125 nm		$I_1 = 5.14 \times 10^{-29}$ kg·m^2
t = 250 nm		$I_2 = 8.06 \times 10^{-28}$ kg·m^2
R_d = 570 nm		
R_r = 4.88 μm		
ψ = 60.2 deg		
$r_{\max}$ = 2.51 μm		

Supplementary Table 1. Measured and calculated parameters for the torsional device studied in this work.

SUPPLEMENTARY NOTE 1: FOURIER TRANSFORMS AND SINGLE SIDED SPECTRAL DENSITIES

We define the Fourier transform of a time-dependent quantity $A(t)$, from which we obtain its spectral representation $A(\Omega)$, as

$$A(\Omega) = \int_{-\infty}^{\infty} A(t) e^{i\Omega t} dt, \tag{1}$$

with the inverse Fourier transform being

$$A(t) = \frac{1}{2\pi} \int_{-\infty}^{\infty} A(\Omega) e^{-i\Omega t} d\Omega. \tag{2}$$

The double-sided spectral density of $A(t)$, $S_{AA}(\Omega)$, is then defined as the Fourier transform of its autocorrelation function $R_{AA}(t) = \langle A(t)A(0)\rangle$ [1, 2]

$$S_{AA}(\Omega) = \int_{-\infty}^{\infty} R_{AA}(t) e^{i\Omega t} dt. \tag{3}$$

This function specifies the intensity of the signal $A(t)$ at a given frequency and is defined for all frequencies, both positive and negative, with the total energy of $A(t)$ being obtained by integrating over the entire spectral domain.

From this double-sided spectral density function, we can also introduce the symmetrized single-sided spectral density [2, 3], defined strictly for positive frequencies as

$$S_A(\Omega) = S_{AA}(\Omega) + S_{AA}(-\Omega). \tag{4}$$

Note that if $S_{AA}(\Omega)$ is an even function with respect to frequency, then equation (4) simply becomes $S_A(\Omega) = 2S_{AA}(\Omega)$. It is this single-sided spectral density that is most often associated with experimentally measured spectra and will therefore be the spectral density we choose to use here.

SUPPLEMENTARY NOTE 2: TORSIONAL MECHANICS

Generally, three-dimensional motion of a mechanical resonator undergoing simple harmonic motion can be described by the displacement function

$$\mathbf{u}(\mathbf{r},t) = x(t)\mathbf{q}(\mathbf{r}), \tag{5}$$

where $x(t)$ is the time-dependent amplitude of motion and $\mathbf{q}(\mathbf{r})$ describes the spatially varying modeshape of the extended resonator structure [1]. Here, we choose to normalize $\mathbf{q}(\mathbf{r})$ such that

it is unitless and has a value of unity at its maximum (*i.e.* $\max|\mathbf{q}(\mathbf{r})| = 1$). In this way, $x(t)$ parametrizes the device's maximum amplitude of motion in units of displacement. A simple, yet effective, model for the dynamics of the system is that of a damped harmonic oscillator, whereby $x(t)$ obeys the equation of motion

$$\ddot{x}(t) + \Gamma\dot{x}(t) + \Omega_{\mathrm{m}}^2 x(t) = \frac{f(t)}{m}, \tag{6}$$

where Γ, Ω_{m}, and m are the mechanical resonator's damping rate, resonant angular frequency, and effective mass, respectively, with $f(t)$ being the external driving force of the system [1].

For the specific case of torsional mechanics, it is more natural to instead characterize the resonator's motion in terms of an angular displacement, $\theta(t)$, from a pre-determined rotation axis, chosen here to be the y-axis. In this case, the displacement of the resonator will be confined to the zx-plane as defined by $\theta(t)h(y)$ (see Supplementary Figure 1a), where we have introduced the scaling function $h(y) \in [0,1]$ that simply determines the magnitude of $\theta(t)$ along the y-axis. Thus, we have assumed the simplified, yet effective, model of torsional mechanics whereby there is no motion in the y-direction [4, 5]. For rigid, linear rotation, the time-dependent displacements $z'(t)$ and $x'(t)$ from an equilibrium point (z,x) will then be given by

$$\begin{aligned} \Delta z(t) &= z'(t) - z = z\left[\cos(\theta(t)h(y)) - 1\right] - x\sin(\theta(t)h(y)) \approx -\theta(t)h(y)x, \\ \Delta x(t) &= x'(t) - x = z\sin(\theta(t)h(y)) + x\left[\cos(\theta(t)h(y)) - 1\right] \approx \theta(t)h(y)z, \end{aligned} \tag{7}$$

where we have used the small angle approximation $\theta(t) \ll 1$, valid for nanomechanical torsional resonators [6, 7]. Using the relations in equation (7), the displacement function in equation (5) takes on the new form

$$\mathbf{u}(\mathbf{r},t) = \theta(t)h(y)z\hat{x} - \theta(t)h(y)x\hat{z} = \theta(t)\mathbf{p}(\mathbf{r}). \tag{8}$$

Here we have introduced a new modeshape function $\mathbf{p}(\mathbf{r}) = h(y)\left(z\hat{x} - x\hat{z}\right)$, which carries units of displacement.

We can now obtain a relationship between $\theta(t)$ and $a(t)$, by equating $\max|\mathbf{u}(\mathbf{r},t)|$ for both the linear and angular motion, given by equations (5) and (8) respectively, resulting in

$$x(t) = r_{\max}\theta(t), \tag{9}$$

where we have used the fact that $\max|\mathbf{p}(\mathbf{r})| = r_{\max} = \sqrt{x_{\max}^2 + z_{\max}^2}$, with $x_{\max}$ ($z_{\max}$) being the maximum extent of the resonator in the x (z) direction. From this relation between linear and

angular displacement, we can derive an equation of motion for $\theta(t)$ from equation (6) as

$$\ddot{\theta}(t)+\Gamma\dot{\theta}(t)+\Omega_{\mathrm{m}}^{2}\theta(t)=\frac{\tau(t)}{I}, \tag{10}$$

where we have now introduced the resonator's effective moment of inertia $I = r_{\mathrm{max}}^{2}m$ and the external time-dependent torque $\tau(t) = r_{\mathrm{max}}f(t)$.

As it is often the case that measurements of mechanical motion are performed in the frequency domain, it is fruitful to Fourier transform equation (10) to obtain a spectral representation of the device's angular motion as

$$\theta(\Omega)=\chi(\Omega)\tau(\Omega), \tag{11}$$

where $\theta(\Omega)$ and $\tau(\Omega)$ are the Fourier transforms of $\theta(t)$ and $\tau(t)$. We have also introduced the generalized angular displacement susceptibility, $\chi(\Omega)$, which relates the angular displacement to the driving torque in frequency space and is given by

$$\chi(\Omega)=\frac{1}{I\left(\Omega_{\mathrm{m}}^{2}-\Omega^{2}-i\Omega\Gamma\right)}. \tag{12}$$

Furthermore, we can use this expression to relate the single-sided angular displacement spectral density, $S_{\theta}(\Omega)$, to the single-sided spectral density of the driving torque as

$$S_{\theta}(\Omega)=|\chi(\Omega)|^{2}S_{\tau}(\Omega). \tag{13}$$

Therefore, if we are able to measure the angular displacement spectrum of a torsional resonator, the torque acting on the system can be inferred via the system's angular displacement susceptibility.

SUPPLEMENTARY NOTE 3: EFFECTIVE MOMENT OF INERTIA

As we shall see, the effective moment of inertia introduced in equation (10) is the geometric parameter that sets the torque sensitivity of a given torsional resonator. An explicit expression for the effective moment of inertia, I, can be determined by investigating the potential energy of the torsional spring, in direct analogy to the method by which one calculates a mechanical mode's effective mass [1]. For a torsional resonator with a position-dependent density $\rho(\mathbf{r})$, the potential energy of a single, infinitesimal element, with volume dV and mass $dm = \rho(\mathbf{r})dV$, will be given by

$$dU=\frac{1}{2}\Omega_{\mathrm{m}}^{2}\theta^{2}(t)|\mathbf{p}(\mathbf{r})|^{2}dm. \tag{14}$$

The total potential energy is then found to be

$$U = \frac{1}{2} I \Omega_{\mathrm{m}}^2 \theta^2(t), \tag{15}$$

with the effective moment of inertia

$$I = \int |\mathbf{p}(\mathbf{r})|^2 dm = \int \rho(\mathbf{r}) h^2(y) \left(x^2 + z^2\right) dV, \tag{16}$$

where the integral performed over the entire volume of the device. We note that if $h(y) \approx 1$ over the extent of the resonator that contains the majority it's mass (physically corresponding to a large, wide torsion paddle), then we can see from equation (16) that $I \approx I_0$, where I_0 is the conventional, geometric moment of inertia of the device. In the following two subsections we will investigate this parameter, using both analytical methods and numerical simulation, for the resonator geometry discussed in this work.

Analytical Model

From equation (16), we see that in order to calculate the effective moment of inertia of a torsional mode, we need to determine it's mechanical modeshape, $\mathbf{p}(\mathbf{r})$, or more specifically, the scaling function, $h(y)$. We begin by developing a simple analytical model for the resonator shown in Supplementary Figure 1b. For this geometry, we treat the system as two coupled, torsional resonators (see Supplementary Figure 2a), one punctuated by the sample disk, the other by the ring segment used to couple to the optical disk, each with it's own torsional spring constant, κ_i, and (geometric) moment of inertia, I_i. For the device considered here, the torsion rods have a simple rectangular cross-section, such that the torsional spring constants will be given by

$$\kappa_i = \frac{\beta t w_i^3 E}{2 l_i (1 + \nu)}, \tag{17}$$

where l_i and w_i are the length and width of the torsion rod, and t, E and ν are the thickness, Young's modulus and Poisson's of the device, while β is a numerical coefficient given by

$$\beta = \frac{1}{3} \left[1 - \frac{192}{\pi^5} \frac{w_i}{t} \sum_{n}^{\infty} \frac{1}{n^5} \tanh\left(\frac{n\pi t}{2 w_i}\right) \right], \tag{18}$$

where n are positive odd integers [4, 8]. Note that we have assumed $t > w_i$, as this is the case for the device studied here.

The moment of inertia of the sample disk is found from its geometry to be

$$I_1 = \frac{m_d}{4}\left(R_d^2 + \frac{t^2}{3}\right), \tag{19}$$

where R_d and $m_d = \rho\pi R_d^2 t$ are the radius and mass of the sample disk, with ρ being the density of the device. On the other hand, the moment of inertia of the ring segment is given by

$$I_2 = \frac{\rho t w_r (2R_r + wr)}{8}\left[\left((R_r + w_r)^2 + R_r^2\right)(\psi - \sin\psi) + \frac{\psi t^2}{3}\right], \tag{20}$$

where w_r, R_r and ψ are the width, radius of curvature and sector angle of the ring segment as shown in Supplementary Figure 1b.

With the above torsional spring constants and moments of inertia, the coupled equations of motion for the device (excluding damping for simplicity) are then given by

$$\begin{aligned} I_1\ddot{\theta}_1 &= -\kappa_1\theta_1 - \kappa_2(\theta_1 - \theta_2), \\ I_2\ddot{\theta}_2 &= -\kappa_2(\theta_2 - \theta_1), \end{aligned} \tag{21}$$

where θ_1 (θ_2) is the angular displacement of the sample disk (ring segment), as demonstrated schematically in Supplementary Figure 2a. Fourier-transforming these equations of motion, such that $\ddot{\theta}_i = -\Omega^2\theta_i$, we can rewrite them in matrix form as $A\Theta = 0$, with A and Θ given by

$$A = \begin{bmatrix} \Omega^2 - \frac{\kappa_1+\kappa_2}{I_1} & \frac{\kappa_2}{I_1} \\ \frac{\kappa_2}{I_2} & \Omega^2 - \frac{\kappa_2}{I_2} \end{bmatrix}, \quad \Theta = \begin{bmatrix} \theta_1 \\ \theta_2 \end{bmatrix}. \tag{22}$$

Solving this system of equations, we find the eigenfrequencies of this coupled system to be

$$\Omega_\pm = \sqrt{\frac{1}{2}\left(\frac{\kappa_2}{I_2} + \frac{\kappa_1+\kappa_2}{I_1}\right) \pm \frac{1}{2}\sqrt{\left(\frac{\kappa_2}{I_2} + \frac{\kappa_1+\kappa_2}{I_1}\right)^2 - \frac{4\kappa_1\kappa_2}{I_1 I_2}}}, \tag{23}$$

where Ω_- (Ω_+) corresponds to the symmetric (antisymmetric) torsional mode. Here we focus on the symmetric mode (as this is the mode examined in this work), which for the experimental parameters given in Supplementary Table 1 has a predicted frequency of $\Omega_-/2\pi = 18.1$ MHz, somewhat larger than the experimentally measured mechanical resonance frequency of $\Omega_\mathrm{m}/2\pi = 14.5$ MHz.

Inserting the analytical expression for the eigenfrequency of the symmetric mode into the system of equations given by equation (22), we obtain θ_2 in terms of θ_1 as

$$\theta_2 = \frac{1}{2}\left(-\frac{I_1}{I_2} + \frac{\kappa_1+\kappa_2}{\kappa_2} + \sqrt{\left(\frac{I_1}{I_2} + \frac{\kappa_1+\kappa_2}{\kappa_2}\right)^2 - \frac{4\kappa_1 I_1}{\kappa_2 I_2}}\right)\theta_1 \approx \frac{\kappa_1+\kappa_2}{\kappa_2}\theta_1, \tag{24}$$

where we have made the experimentally relevant approximation $I_1 \ll I_2$.

We can now use the relative angular displacements of the sample disk and ring segments given in equation (24) to determine the modeshape scaling function $h(y)$. To do this, we assume the simplest imaginable torsional modeshape [4, 5], where the mechanical device is rigidly clamped at one end ($\theta = 0$), with the angle of deflection increasing linearly along the torsion rods, while remaining constant over both the sample disk and ring segment. In this case, $h(y)$ is given by the piecewise function

$$h(y) = \begin{cases} \frac{\kappa_2}{\kappa_1+\kappa_2}\frac{y}{l_1}, & y \in A \\ \frac{\kappa_2}{\kappa_1+\kappa_2}, & y \in B \\ \frac{\kappa_1}{\kappa_1+\kappa_2}\frac{y-l_1-2R_d}{l_2} + \frac{\kappa_2}{\kappa_1+\kappa_2}, & y \in C \\ 1, & y \in D. \end{cases} \tag{25}$$

A plot of this function using the measured/calculated device parameters (as given in Supplementary Table 1) can be seen in Supplementary Figure 2b, where we have also defined the regions *A*-*D*.

Finally, by inputting equation (25) into equation (16), we can determine the effective moment of inertia for this simple analytical model to be

$$\begin{aligned} I &= \left(\frac{\theta_1}{\theta_2}\right)^2 \frac{I_{\kappa_1}}{3} + \left[1 + \frac{\theta_1}{\theta_2} + \left(\frac{\theta_1}{\theta_2}\right)^2\right]\frac{I_{\kappa_2}}{3} + \left(\frac{\theta_1}{\theta_2}\right)^2 I_1 + I_2 \\ &\approx \left(\frac{\kappa_2}{\kappa_1+\kappa_2}\right)^2 \frac{I_{\kappa_1}}{3} + \left[1 + \frac{\kappa_2}{\kappa_1+\kappa_2} + \left(\frac{\kappa_2}{\kappa_1+\kappa_2}\right)^2\right]\frac{I_{\kappa_2}}{3} + \left(\frac{\kappa_2}{\kappa_1+\kappa_2}\right)^2 I_1 + I_2 \approx I_2, \end{aligned} \tag{26}$$

where I_{κ_1} and I_{κ_2} are the geometric moments of inertia corresponding to the two torsion rods with spring constants κ_1 and κ_2 ($I_{\kappa_i} = \rho l_i w_i t(w_i^2 + t^2)/12$), and we have again used the experimentally valid approximation that $I_2 \gg I_1, I_{\kappa_1}, I_{\kappa_2}$. For the device studied here, the analytical model predicts an effective moment of inertia $I = 807$ fg$\cdot\mu$m^2.

Finite Element Method Simulation

While the analytical model of the previous section allows for a qualitative understanding of the torsional modeshape of our resonator, a much more accurate modeshape, and therefore effective moment of inertia, can be determine using finite element method (FEM) simulations. An example of such a modeshape generated using COMSOL can be seen in the inset of Figure 1d of the main text. From this simulated modeshape, the effective moment of inertia of the device can be

calculated numerically using equation (16), for which we find a value of $I = 774$ fg$\cdot\mu$m^2. It is this value that we use to calculate the torque sensitivities in the main text.

Furthermore, the scaling function $h(y)$ extracted from the FEM simulation is compared to its analytically determined counterpart given by equation (25) in Supplementary Figure 2b, highlighting the deviation of the analytical model from the numerical one. This disparity is likely due to the fact that the analytical model ignores the resonator's support structure, as well as the elasticity of the material. As such, the analytical model overshoots the scaling function in the critical region of the ring segment, predicting a larger effective moment of inertia than the numerical model.

SUPPLEMENTARY NOTE 4: LIMITS ON CONTINUOUS LINEAR TORQUE MEASUREMENTS

The limit on the sensitivity one can obtain by performing a continuous linear torque measurement using a mechanical resonator is set by the noise that will inevitably creep into the system, contaminating the measurement. Minimizing this noise will allow the system to resolve smaller torques, leading to an increase in sensitivity.

In order to determine the limiting torque noise, we consider the total angular displacement noise spectrum

$$S_\theta(\Omega) = S_\theta^{\mathrm{qu}}(\Omega) + S_\theta^{\mathrm{imp}}(\Omega) + S_\theta^{\mathrm{ba}}(\Omega), \tag{27}$$

which is a combination of the intrinsic noise due to the thermal and quantum fluctuations of the mechanical element, $S_\theta^{\mathrm{qu}}(\Omega)$, along with the imprecision, $S_\theta^{\mathrm{imp}}(\Omega)$, and back-action, $S_\theta^{\mathrm{ba}}(\Omega)$, noise spectra generated by the measurement apparatus.

The intrinsic angular noise spectrum can be determined using the (quantum) fluctuation-dissipation theorem [9], expressed mathematically for the single-sided angular displacement as

$$S_\theta^{\mathrm{qu}}(\Omega) = 4\hbar\left(\langle n\rangle + 1/2\right)\mathrm{Im}\{\chi(\Omega)\} = 4\hbar\Omega\Gamma I\left(\langle n\rangle + 1/2\right)|\chi(\Omega)|^2. \tag{28}$$

Comparing this expression to equation (13), we can immediately identify the intrinsic quantum torque spectrum as $S_\tau^{\mathrm{qu}}(\Omega) = 4\hbar\Omega\Gamma I\left(\langle n\rangle + 1/2\right)$, where $\langle n\rangle$ is the phonon occupation of the torsional mode. For the case of thermal equilibrium with a bath at temperature T, the phonon occupation is given by the Bose-Einstein occupation factor $\langle n\rangle = \bar{n}_{\mathrm{th}} = (e^{\hbar\Omega/k_BT} - 1)^{-1}$. Note that in equation (28) there is an addition of one-half to this thermal occupation, corresponding to the ground state motion of the mechanical resonator. In the high-temperature limit, $\langle n\rangle \approx k_BT/\hbar\Omega \gg 1$, this

ground state contribution can be neglected and $S_\tau^{\mathrm{qu}}(\Omega)$ reduces to the familiar classical white-noise torque spectrum of $S_\tau^{\mathrm{cl}} = 4k_BT\Gamma I$ [1].

Likewise, one can express the back-action angular noise spectrum as $S_\theta^{\mathrm{ba}}(\Omega) = |\chi(\Omega)|^2 S_\tau^{\mathrm{ba}}(\Omega)$, where we have now introduced a back-action torque noise spectrum, $S_\tau^{\mathrm{ba}}(\Omega)$. In general, this back-action torque spectrum, as well as the angular imprecision noise spectrum, $S_\theta^{\mathrm{imp}}(\Omega)$, will contain contributions from both classical technical noise and fundamental quantum noise, with the product of the two spectra obeying the Heisenberg uncertainty relation (for single-sided spectra) [2, 3, 9]

$$S_\tau^{\mathrm{ba}}(\Omega) S_\theta^{\mathrm{imp}}(\Omega) \geq \hbar^2. \tag{29}$$

Note that equality in equation (29) corresponds to quantum-limited measurement noise (*i.e.* no classical noise).

We can now determine an equivalent torque noise spectrum from equation (27) using the angular susceptibility of the system as

$$S_\tau(\Omega) = \frac{S_\theta(\Omega)}{|\chi(\Omega)|^2} = 4\hbar\Omega\Gamma I \left(\langle n\rangle + n_{\mathrm{imp}}(\Omega) + n_{\mathrm{ba}}(\Omega) + 1/2\right), \tag{30}$$

It is this torque noise spectrum that sets the minimum resolvable torque, and hence the sensitivity, of our system. In equation (30), we have introduced the equivalent noise quanta due measurement imprecision and back-action, $n_{\mathrm{imp}}(\Omega) = S_\theta^{\mathrm{imp}}(\Omega)/4\hbar\Omega\Gamma I|\chi(\Omega)|^2$ and $n_{\mathrm{ba}}(\Omega) = S_\theta^{\mathrm{ba}}(\Omega)/4\hbar\Omega\Gamma I|\chi(\Omega)|^2$, whose product can be found from equation (29) to obey

$$n_{\mathrm{imp}}(\Omega) n_{\mathrm{ba}}(\Omega) \geq \frac{1}{16\Omega^2\Gamma^2 I^2 |\chi(\Omega)|^2}, \tag{31}$$

with equality again corresponding to quantum-limited measurement noise. Note that at the mechanical resonance frequency, equation (31) reduces to the familiar form $n_{\mathrm{imp}}(\Omega_{\mathrm{m}}) n_{\mathrm{ba}}(\Omega_{\mathrm{m}}) \geq 1/16$ [3].

We now interest ourselves in determining the minimum possible torque noise spectrum as allowed by quantum mechanics. To do this, we first look to minimize the added measurement noise of our system

$$S_\theta^{\mathrm{add}}(\Omega) = S_\theta^{\mathrm{imp}}(\Omega) + S_\theta^{\mathrm{ba}}(\Omega) = S_\theta^{\mathrm{imp}}(\Omega) + |\chi(\Omega)|^2 S_\tau^{\mathrm{ba}}(\Omega). \tag{32}$$

By taking equality in the Heisenberg uncertainty relation of equation (29), we find the optimal measurement noise spectra of $S_\theta^{\mathrm{imp}}(\Omega) = S_\theta^{\mathrm{ba}}(\Omega) = \hbar|\chi(\Omega)|$, or equivalently, $S_\tau^{\mathrm{ba}}(\Omega) = \hbar/|\chi(\Omega)|$, corresponding to the so-called standard quantum limit (SQL) of continuous position measurement [2, 9]. Furthermore, by tuning to the mechanical resonance frequency, $|\chi(\Omega)|$ is maximized,

thus minimizing the torque noise $S_\tau(\Omega)$ in equation (30) with respect to frequency. Returning to our effective quanta notation, we find that $n_{\mathrm{imp}}(\Omega_{\mathrm{m}}) = n_{\mathrm{ba}}(\Omega_{\mathrm{m}}) = 1/4$ at the SQL, such that the minimized torque noise is found to be

$$S_\tau^{\mathrm{SQL}} = S_\tau^0(\langle n\rangle + 1), \tag{33}$$

where $S_\tau^0 = 4\hbar\Omega_{\mathrm{m}}\Gamma I$ is the fundamental torque noise associated with the continuous monitoring of a mechanical resonator in its quantum ground state at the SQL. It is this zero-point torque spectrum that sets the quantum limit for the minimum resolvable torque for a given device, half of which arises from the zero-point motion of the resonator, the other half from the quantum-limited measurement noise at the SQL, whereas the limit at finite temperature will be given by the quantity in equation (33).

SUPPLEMENTARY NOTE 5: EFFECT OF OPTOMECHANICAL BACK-ACTION COOLING

One method by which one might think to decrease the minimum resolvable torque spectrum as given by equation (33) is to reduce the phonon occupancy of the mechanical resonator using a cold damping method, such as optomechanical back-action cooling (OBC) [10, 11]. Unfortunately, as we will see below, the minimum resolvable torque spectrum in fact increases.

In OBC, photons trapped in an optical cavity impart a dynamical radiation pressure force on the mechanical resonator, increasing its intrinsic linewidth by an amount Γ_{OM} and reducing its average phonon occupancy according to

$$\langle n\rangle = \frac{\Gamma\bar{n}_{\mathrm{th}} + \Gamma_{\mathrm{OM}}\bar{n}_{\mathrm{min}}}{\Gamma + \Gamma_{\mathrm{OM}}}, \tag{34}$$

where $\bar{n}_{\mathrm{min}}$ is the minimum obtainable average phonon occupancy using this method [10, 11] ($\bar{n}_{\mathrm{min}} = \kappa/4\Omega_{\mathrm{m}}$ for $\kappa \gg \Omega_{\mathrm{m}}$, whereas $\bar{n}_{\mathrm{min}} = \kappa^2/16\Omega_{\mathrm{m}}^2$ in the sideband resolved case of $\kappa \ll \Omega_{\mathrm{m}}$, with κ being the linewidth of the optical cavity). Note that for $\Gamma_{\mathrm{OM}} = 0$ (*i.e.* no optomechanical damping), $\langle n\rangle = \bar{n}_{\mathrm{th}}$ and thermal equilibrium is restored.

Inputting equation (34) into equation (33), we obtain the minimum resolvable torque associated

with OBC as

$$\begin{aligned} S_\tau^{\text{obc}} &= 4\hbar\Omega_{\text{m}} I(\Gamma+\Gamma_{\text{OM}})\left(\frac{\Gamma\bar{n}_{\text{th}}+\Gamma_{\text{OM}}\bar{n}_{\text{min}}}{\Gamma+\Gamma_{\text{OM}}}+1\right) \\ &= 4\hbar\Omega_{\text{m}} I(\Gamma\bar{n}_{\text{th}}+\Gamma_{\text{OM}}\bar{n}_{\text{min}})+4\hbar\Omega_{\text{m}} I(\Gamma+\Gamma_{\text{OM}}) \\ &= 4\hbar\Omega_{\text{m}}\Gamma I(\bar{n}_{\text{th}}+1)+4\hbar\Omega_{\text{m}}\Gamma_{\text{OM}} I(\bar{n}_{\text{min}}+1) \\ &= S_\tau^{\text{SQL}}+S_\tau^{\text{OM}} \geq S_\tau^{\text{SQL}}, \end{aligned} \tag{35}$$

where $S_\tau^{\text{OM}} = 4\hbar\Omega_{\text{m}}\Gamma_{\text{OM}} I(\bar{n}_{\text{min}}+1)$ is the contribution to the minimum resolvable torque spectrum due to optomechanical back-action. Note that the inequality in the last line of equation (35) arises due to the fact that $S_\tau^{\text{OM}} \geq 0$ for optomechanical damping (where $\Gamma_{\text{OM}} \geq 0$), with equality corresponding to $\Gamma_{\text{OM}} = 0 \Rightarrow S_\tau^{\text{OM}} = 0$. Therefore, one can see that while the phonon occupancy of the mechanical mode decreases, there is in fact an increase in the minimum resolvable torque spectrum. Physically, this can be understood due to the fact that any apparent reduction in the minimum resolvable torque spectrum attributed to a decrease in the phonon occupancy of the resonator is nullified by the corresponding increase in the mechanical damping rate.

SUPPLEMENTARY NOTE 6: OPTOMECHANICAL TORQUE TRANSDUCTION

In this work, torsional motion is transduced optomechanically, whereby the torsional motion of the mechanical resonator is coupled to photons confined in an optical cavity. Here, the coupling is dispersive in the sense that the resonance frequency of the optical cavity $\omega_{\text{c}}(\theta(t))$ is a function of the angular motion $\theta(t)$, which can be expanded to first order as

$$\omega_{\text{c}}(\theta(t)) \approx \omega_c + G_\theta \theta(t), \tag{36}$$

where ω_c is the unperturbed cavity resonance frequency and $G_\theta = d\omega_{\text{c}}/d\theta$ is the angular optomechanical coupling coefficient. Note that we can use equation (9) to relate G_θ to the standard optomechanical coupling coefficient for linear motion, $G_x = d\omega_{\text{c}}/dx$, as $G_\theta = r_{\text{max}} G_x$.

This mechanically induced optical frequency shift will result in amplitude and phase fluctuations in the optical signal transmitted through the cavity, both of which can be detected (using either a tuned-to-slope or homodyne measurement) as an AC voltage signal on a photodiode. This time-varying signal can then be converted into a voltage spectral density given by

$$S_V(\Omega) = \alpha S_\theta(\Omega), \tag{37}$$

from which we can infer the angular displacement spectral density $S_\theta(\Omega)$ by determining the properly calibrated conversion coefficient α, which has units of V^2/rad^2. In this work, this is done by thermomechanically calibrating the voltage spectrum using the method of Ref. [1].

SUPPLEMENTARY NOTE 7: LOW DUTY CYCLE MEASUREMENTS

In the absence of helium exchange gas, the mechanical resonator is easily heated by injected optical power. To circumvent this heating, low-duty cycle measurements are performed. The optical power is adjusted using a voltage-controlled variable optical attenuator—labeled in Supplementary Figure 3 as control—and is pulsed on for 20 ms, as dictated by the mechanical damping rate at low temperatures, $\Gamma/2\pi = 340$ Hz. We then wait 120 s without injecting any light, to allow for re-thermalization to the dilution refrigerator temperature. The AC spectrum from 100 of these pulse sequences is averaged to extract each of the data sets shown in Figure 3 of the main text.

SUPPLEMENTARY REFERENCES

[1] Hauer, B. D., Doolin, C., Beach, K. S. D. & Davis, J. P. A general procedure for thermomechanical calibration of nano/micro-mechanical resonators. *Ann. Phys.* **339**, 181-207 (2013).

[2] Clerk, A. A., Devoret, M. H., Girvin, S. M., Marquardt, F. & Schoelkopf, R. J. Introduction to quantum noise, measurement, and amplification. *Rev. Mod. Phys.* **82**, 1155-1208 (2010).

[3] Wilson, D. J., Sudhir, V., Piro, N., Schilling, R., Ghadimi, A. & Kippenberg, T. J. Measurement-based control of a mechanical oscillator at its thermal decoherence rate. *Nature* **524**, 325-329 (2014).

[4] Timoshenko, S. P. & Goodier, J. N. Theory of Elasticity. Third ed., McGraw-Hill (1970).

[5] Sokolnikoff, I. S. Mathematical Theory of Elasticity. Second ed., McGraw-Hill (1956).

[6] Kim, P. H., Doolin, C., Hauer, B. D., MacDonald, A. J. R., Freeman, M. R., Barclay, P. E. & Davis, J. P. Nanoscale torsional optomechanics. *Appl. Phys. Lett.* **102**, 053102 (2013).

[7] Wu, M., Hryciw, A. C., Healey, C., Lake, D. P., Jayakumar, H., Freeman, M. R., Davis, J. P. & Barclay, P. E. Dissipative and dispersive optomechanics in a nanocavity torque sensor. *Phys. Rev. X* **4**, 021052 (2014).

[8] Bao, M.-H. Analysis and Design Principles of MEMS Devices. First ed., Elsevier (2005).

[9] Braginsky, V. B. & Khalili, F. Y. Quantum Measurement. First ed., Cambridge (1992).

[10] Wilson-Rae, I., Nooshi, N., Zwerger, W. & Kippenberg, T. J. Theory of Ground State Cooling of a Mechanical Oscillator Using Dynamical Backaction. *Phys. Rev. Lett.* **99**, 093901 (2007).

[11] Marquardt, F., Chen, J. P., Clerk. A. A. & Girvin, S. M. Quantum Theory of Cavity-Assisted Sideband Cooling of Mechanical Motion. *Phys. Rev. Lett.* **99**, 093902 (2007).
